# Whooping cough in the most vulnerable: A case series of pertussis in infants younger than three months in Qatar

**DOI:** 10.5339/qmj.2025.18

**Published:** 2025-03-04

**Authors:** Mohammad Alesali, Mohammad Elhamidi

**Affiliations:** ^1^Pediatric Department, Al Khor Hospital, Hamad Medical Corporation, Doha, Qatar; ^2^Clinical Science Department, College of Medicine, Qatar University, Doha, Qatar *Correspondence: Mohammad Alesali. Email: malesali@hamad.qa

**Keywords:** Infants, pediatrics, pertussis, whooping cough, prevention, maternal vaccination

## Abstract

**Background:**

Pertussis, a highly contagious disease, has made a resurgence following the easing of COVID-19 pandemic restrictions. Infants under three months old are particularly vulnerable due to their immature immune systems and lack of protective vaccination.

**Methods:**

This study presents a case series of four infants, aged one to three months, who initially presented with nonspecific respiratory symptoms at Alkhor Hospital between January and June 2024. Subsequent diagnostic testing confirmed pertussis in all four cases. It is noteworthy that all cases involved were previously healthy infants with no underlying health conditions. Additionally, none of the mothers had received the Tdap vaccine during pregnancy.

**Results:**

All infants required hospitalization, with one being admitted to the PICU for eight days. Ultimately, all four infants made a full recovery.

**Conclusion:**

Pertussis remains a significant cause of morbidity and mortality in infants under three months of age. Given the potential for severe complications and the burden it places on the healthcare system during outbreaks, it is crucial to emphasize preventive measures such as maternal vaccination.

## Introduction

*Bordetella pertussis* is a highly contagious Gram-negative bacterium that was first identified in 1906. The bacterium is transmitted through respiratory droplets, with an incubation period that typically lasts from one to three weeks.^1^ The classical clinical presentation of pertussis is characterized by whooping sound followed by vomiting. Vaccinated children, infants, and children over the age of 10 may not exhibit whooping cough.^2^

**Figure 1.**
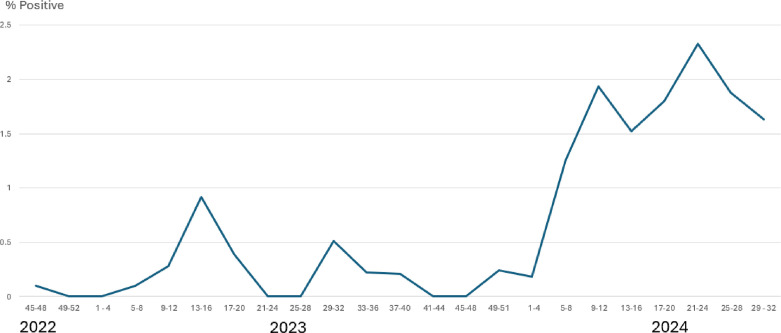
Chart showing the rolling four-week ratio of positive pertussis respiratory PCR (polymerase chain reaction) tests conducted in Hamad Medical Corporation from late 2022 up to week 32 of 2024. A dramatic surge in the ratio of positive pertussis results is observed in 2024 when compared to 2023. (This chart was kindly provided with permission to publish by Dr. Peter Valentine Coyle, Head of Virology Department in Hamad Medical Corporation.)

Pertussis was a serious cause of morbidity and mortality before the vaccination era. The widespread use of the whole-cell vaccine in the second half of the 20th century led to a decline in both the incidence and mortality associated with pertussis. Specifically, the global mortality rates among children under five years of age decreased from 4 million cases per year in 1950, for a population of 2.5 billion, to less than 100,000 cases in 2020, for a population of 7.5 billion.^3^ However, there has been a resurgence of pertussis following the implementation of acellular pertussis vaccines with milder clinical presentations. This increase may be attributed to the shorter duration of immunity offered by the new vaccine compared to the older one, combined with insufficient booster vaccinations among both adults and adolescents.^3^ The social distancing measures implemented during the COVID-19 pandemic in 2020 led to a decrease in new cases of pertussis,^4–6^ followed by a global rebound after these restrictions were lifted. In China, there was a 45-fold increase in the reported cases of pertussis during the first four months of 2024, compared to the previous 12 months of 2023. Similar trends were reported in other countries.^7^ Qatar also seems to be experiencing a similar surge in pertussis cases during the same period (Figure 1).

Infants under three months of age, who have not yet received complete vaccinations, are particularly vulnerable to pertussis worldwide. In certain countries experiencing outbreaks, the incidence rate may exceed 1,000 cases per 100,000 infants, with the majority of fatalities, hospitalizations, and admissions to intensive care units occurring in this age group.^8^

This case series reports four cases of infants younger than three months old who were evaluated in the Pediatric Emergency Department at Alkhor Hospital over six months in 2024. These infants initially presented with nonspecific respiratory symptoms, which were later confirmed to be due to pertussis.

## Case Presentation

### Case 1

A female infant, aged 51 days old and weighing 4.5 kg, was born at full term without any complications. She was brought to a primary healthcare center by her parents, having experienced a cough, runny nose, and mild fever for the past two days. The infant's feeding and sleep patterns remained unchanged. Vaccination was up to date, but the DTaP vaccine was not yet due. Additionally, the mother did not receive the Tdap vaccine during her pregnancy. The infant's older sibling had similar complaints recently. On examination, vital signs were normal, with a temperature of 37.6°C, a heart rate of 136 beats per minute, a respiratory rate of 38 breaths per minute, and an oxygen saturation of 98%. The infant appeared generally well, with the exception of exhibiting rhinorrhea. Nasal normal saline drops were prescribed, and the patient was discharged.

Two days later, the infant returned to the pediatric emergency department due to a worsening cough. The parents reported that the infant was breathing fast. However, on assessment, the patient did not exhibit tachypnea (respiratory rate 44 breaths per minute) or any signs of respiratory distress. The infant was afebrile and maintained normal oxygen saturation on room air. Auscultation of the chest revealed good bilateral air entry with bilateral wheezing and crackles. The patient was started on intravenous fluids, and laboratory tests were initiated, leading to admission for further management of acute bronchiolitis.

The complete blood count revealed a normal white blood cell count (10.8 × 10^3^/μL), with a normal absolute lymphocyte count (6.23 × 10^3^/μL). Both red blood cell indices and platelet count were within the reference ranges. Nasopharyngeal swab polymerase chain reaction (PCR) was positive for mycoplasma pneumonia, respiratory syncytial virus, and *Bordetella pertussis*. Blood cultures and other laboratory tests were unremarkable, and no nasopharyngeal secretion culture was obtained.

During the hospitalization, the infant was administered nasal saline drops, paracetamol as needed for a single temperature spike of 38°C, and oral azithromycin. Clinical status showed improvement, characterized by the resolution of respiratory distress and a normal chest examination. The infant was discharged after 24 hours, continuing treatment with nasal saline drops, paracetamol, and a five-day course of oral azithromycin. The parents were advised to return to the pediatric emergency department if any signs of respiratory distress developed.

**Figure 2.**
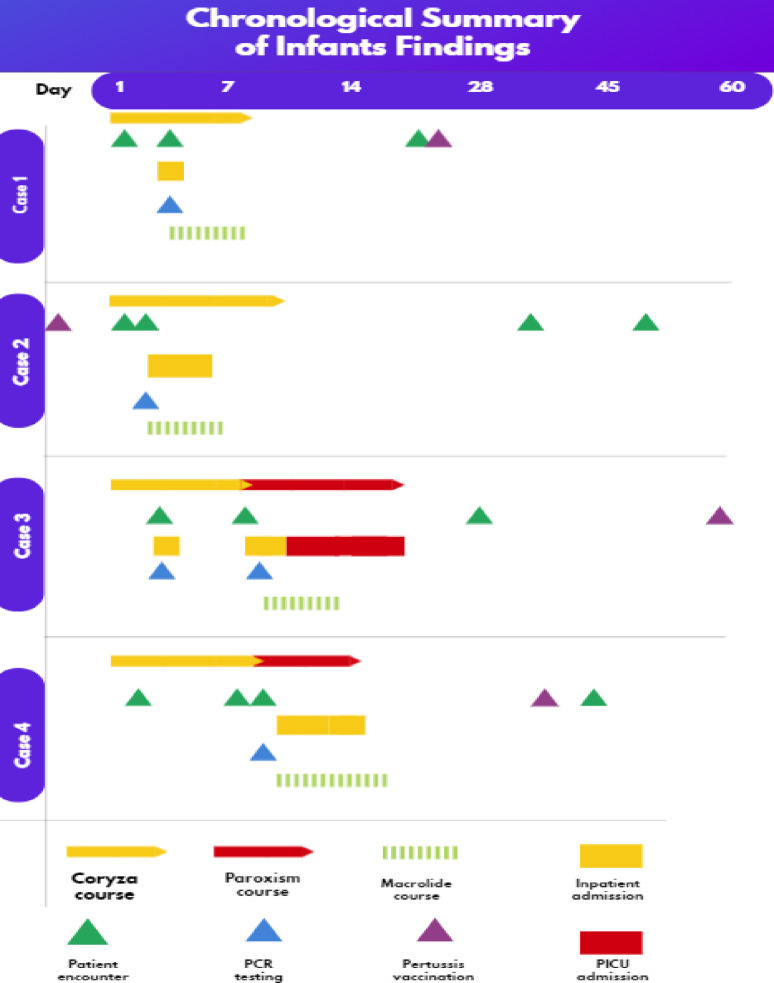
Timeline providing an approximate chronological overview of the medical history of the infants, including symptoms, clinical presentations, diagnostic PCR results, hospitalizations, and vaccination status.

During the two-week follow-up, the infant was asymptomatic and had a normal physical examination. Routine two-month vaccinations were administered two days later. Key features of this case are summarized in Table 1 and Figure 2.

### Case 2

A male infant, 73 days old and weighing 5.4 kg, was born at full term without complications. He presented to the pediatric emergency department at Alkhor Hospital with a two-day history of cough and rhinorrhea, but without fever. Five days earlier, the infant had received the routine two-month vaccinations, including the hexa vaccine. Notably, his mother did not receive the Tdap vaccine during her pregnancy. The physical examination was unremarkable except for rhinorrhea. Consequently, the infant was discharged with a prescription for nasal saline drops.

The following day, PCR results were positive for both *Bordetella pertussis* and human rhino/enterovirus. As a result, the patient was admitted for observation, intravenous fluids, and laboratory tests. The white blood cell count was slightly elevated at 15.8 × 10^3^/μL, with a mild lymphocytosis (12.2 × 10^3^/μL). All other laboratory parameters, including platelet count, red blood cell indices, C-reactive protein, electrolytes, and renal and liver function tests were within normal limits. No nasopharyngeal secretion culture was obtained.

Treatment included the administration of azithromycin and hypertonic saline nebulization. The patient was discharged after three days on oral azithromycin, with a follow-up scheduled.

The infant returned for a follow-up visit at one and one-and-a-half months after the initial presentation. Although the cough frequency had decreased, it persisted during both visits. Key features of this case are summarized in Table 1 and Figure 2.

### Case 3

A 40-day-old female infant, weighing 4.5 kg, was delivered at full term without complications. She was brought to the pediatric emergency department at Alkhor Hospital due to a three-day history of noisy breathing and coughing episodes, accompanied by brief pauses in breathing and bluish discoloration of the lips. The infant had no fever or other symptoms. Vaccination was up to date, but the DTaP vaccine was not yet due. Her mother did not receive the Tdap vaccine during her pregnancy. There was a reported exposure to a sick individual with respiratory symptoms at home. Vital signs were within normal limits, including heart rate, respiratory rate, and oxygen saturation. The initial examination revealed no signs of respiratory distress, with clear breath sounds noted in both lungs with occasional crackles, and normal findings in the rest of the physical examination.

The infant was admitted for observation and was provided with intravenous fluids, along with normal saline nebulization every two hours. The chest X-ray was normal, showing no evidence of peribronchial cuffing. The complete blood count, which included a white blood cell count of 14.6 × 10^3^/μL, an absolute lymphocyte count of 10.5 × 10^3^/μL, as well as differential, red blood cell indices, and platelet counts, were all found to be within normal limits. Blood cultures, C-reactive protein levels, electrolytes, renal and liver function tests were unremarkable. A nasopharyngeal respiratory PCR test was positive for human rhino/enterovirus. The infant was discharged against medical advice with instructions to follow up within 24 hours.

**Figure 3.**
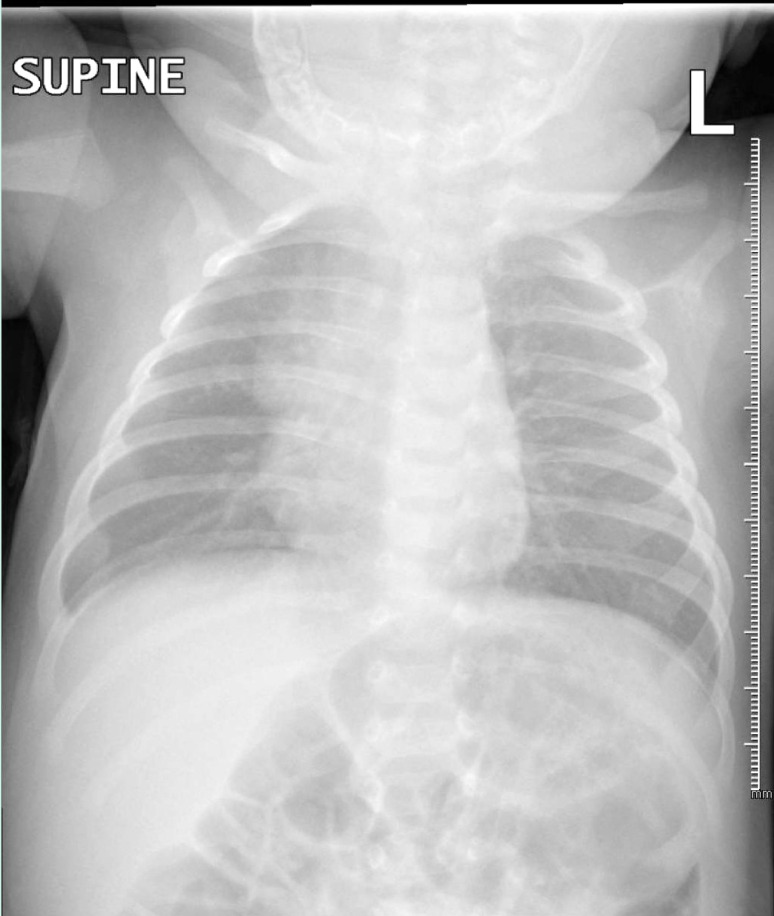
Chest X-ray was performed before the admission of the third case to the floor. It reveals prominent bronchovascular markings in the perihilar regions with mild bilateral peribronchial thickening. Additionally, bilateral scattered infiltrates in the perihilar parenchyma are noted. The cardiothymic contour is age-appropriate, and the costophrenic angles are clear.

Five days later, the patient returned with an increased frequency of coughing episodes, accompanied by bluish discoloration of the face, vomiting, and decreased feeding. On initial assessment, vital signs were stable, and the infant was active. A physical examination revealed good bilateral air entry with fine crackles. The infant was diagnosed with acute bronchiolitis and treated with normal saline nebulization every four hours, intravenous fluids, and monitoring. Blood tests were normal, except for elevated platelet count (719 × 10^3^/μL). A chest X-ray showed increased bronchovascular markings in the perihilar regions with mild peribronchial thickening (Figure 3). Oxygen therapy was initiated via nasal cannula at 1 L/min due to decreased oxygen saturation up to 92% on room air.

The infant was admitted to the pediatric ward for continued treatment of acute rhinovirus bronchiolitis. On the second day of hospitalization, she developed tachypnea at a rate of 55 breaths per minute, accompanied by visible subcostal retractions. To prevent the risk of aspiration, nasogastric tube feeding was initiated. Subsequently, the infant experienced episodes of frequent coughing with cyanosis. On the same day, there was an episode of apnea lasting approximately 35 seconds, accompanied by desaturation requiring manual ventilation using a bag-valve mask. A significant amount of mucus was suctioned from the nasopharynx. Azithromycin was started due to suspected pertussis. A repeat respiratory PCR test confirmed the presence of *Bordetella pertussis* in addition to the previously detected human rhino/enterovirus infections. No nasopharyngeal secretion culture was obtained.

**Figure 4.**
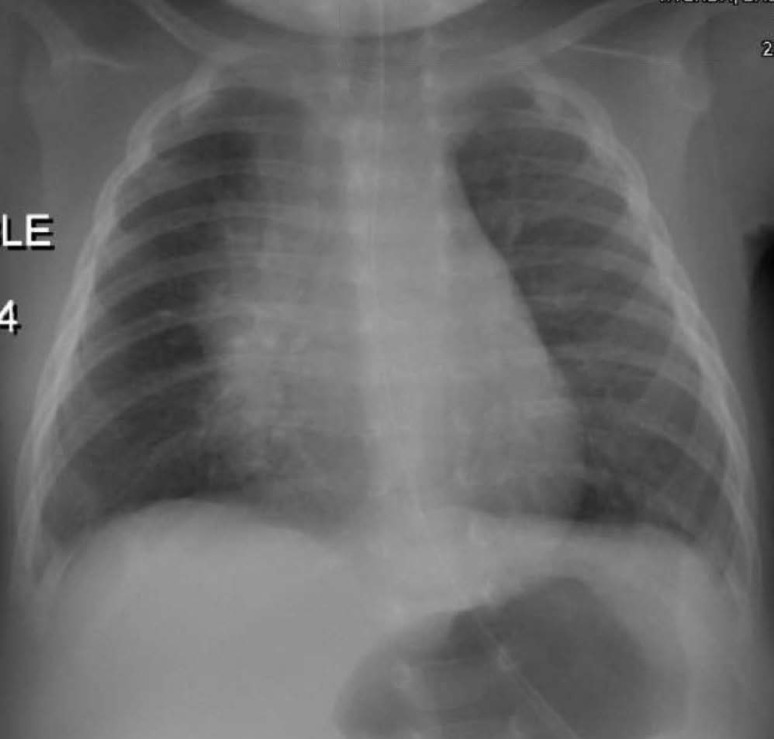
Chest X-ray was performed following the admission of the third case to the PICU. In comparison to the previous chest X-ray, there is a progression of retrocardiac and left lower lung zone infiltrations.

The infant was subsequently transferred to the pediatric intensive care unit (PICU) for closer monitoring and advanced respiratory support if needed. Initial management in the PICU included the administration of normal saline nebulization, suctioning, azithromycin, and nasogastric feeding. Respiratory support was increased to a high-flow nasal cannula set at 7 L/min with 30% oxygen. A chest X-ray showed progression of infiltrations behind cardiac shadow and in the left lower lung zone (Figure 4).

Over the next several days, the infant experienced multiple episodes of decreased oxygen saturation and heart rate, accompanied by coughing. Nevertheless, the infant's overall condition gradually improved, requiring less respiratory support and transitioning to oral feeding.

On the eighth day, the infant was discharged from the PICU with a prescription for normal saline nasal drops and instructions to seek immediate medical care if any concerns arose. A follow-up visit one week later showed a stable condition, and the infant received two-month vaccinations one month after discharge. Key features of this case are summarized in Table 1 and Figure 2.

### Case 4

A male infant, 58 days old and weighing 5.7 kg, was born from a full-term, uneventful pregnancy. He was brought to the primary health care center with a two-day history of cough, rhinorrhea, and respiratory distress. Vaccination was up to date, but the DTaP vaccine was not yet due. His mother did not receive the Tdap vaccine during her pregnancy. A family member had recently experienced an upper respiratory tract infection. On examination, vital signs, including temperature, respiratory rate, heart rate, and oxygen saturation, were all within normal limits, and the infant had no signs of respiratory distress. A chest examination revealed good bilateral air entry with mild bilateral wheezing, while the rest of the examination was unremarkable. The infant was subsequently referred to the pediatric emergency department at Al Sadd Hospital, where he was diagnosed with acute bronchiolitis and discharged with a prescription for nasal normal saline drops.

Five days later, the patient returned to the primary health center with a persistent cough, although there was no fever or rhinorrhea. Vitals were within normal limits, and the physical examination showed no signs of respiratory distress, although mild bilateral wheezing persisted. Consequently, the infant was discharged with the addition of a salbutamol inhaler.

Subsequently, three days after the last visit, the patient was brought to the pediatric emergency department at Alkhor Hospital due to episodes of paroxysmal coughing that were accompanied by cyanosis. An initial assessment revealed stable vital signs and no respiratory distress. The chest examination was unremarkable. During the observation period in the emergency department, the infant experienced two episodes of paroxysmal coughing with cyanosis. As a result, oral clarithromycin was initiated, and the infant was admitted for further management. A complete blood count revealed that the WBC (10.1 × 10^3^/μL), the absolute lymphocyte counts (7.4 × 10^3^/μL), the differential, red blood cell indices, and platelet counts were within normal limits. A nasopharyngeal PCR was positive for *Bordetella pertussis*. No nasopharyngeal secretion culture was obtained. Blood cultures and other laboratory tests, including C-reactive protein, electrolytes, and renal and liver function tests, were unremarkable.

The infant was hospitalized for five days, during which he was on oral clarithromycin, hypertonic saline nebulization, and intravenous fluids. Throughout the hospitalization, there was a noted decrease in both the frequency and severity of spasmodic coughing episodes. Chest radiography results were normal. The infant was discharged from the hospital on the fifth day of admission, as he exhibited a stable condition with normal vital signs, no fever, and good feeding. The parents were advised to complete the seven-day course of oral clarithromycin.

Twenty days after discharge, the infant received routine two-month vaccinations, followed by an asymptomatic follow-up evaluation with a normal physical examination 10 days later. Key features of this case are summarized in Table 1 and Figure 2.

## Discussion

### Clinical features of pertussis

The classical presentation of pertussis is predominantly observed in children under the age of 10 years who have not been vaccinated against the disease. The illness progresses through three different stages. The initial **catarrhal stage** resembles a viral upper respiratory infection, characterized by cough, watery rhinorrhea, and lacrimation. Fever, if present, is typically mild or absent. Symptoms generally worsen over a period of one to two weeks. In the **paroxysmal stage**, the cough manifests as a series of prolonged episodes with minimal or no respiratory effort, potentially resulting in cyanosis and bulging of the neck veins. The inspiratory effort following cough makes a noise that is called whooping. Additionally, post-tussive vomiting is more frequently observed in infants under one year of age. This stage can be complicated by various conditions, including pneumonia, seizures, intracranial hemorrhage, rib fractures, otitis media, or even death. During episodes of paroxysms, the patient appears exhausted, although they seem well outside these episodes. This condition typically persists for a duration of two to eight weeks and then gradually decreases. The cough tends to subside gradually during the **convalescent stage**, which may extend over several weeks or even months.^2^

Children over the age of 10 years may either be asymptomatic or exhibit mild symptoms. Vaccinated children generally experience less severe and shorter-lasting symptoms.^2^

Infants younger than three months often do not show the characteristic features and are more prone to complications. A study by Nieves et al. revealed that during the pertussis outbreak in California in 2010, the majority of hospitalizations and all reported fatalities were among infants under three months of age. In this age group, pertussis was overlooked in earlier medical consultations. The study identified several characteristics that should prompt clinicians to consider pertussis rather than a viral infection. These characteristics include a paroxysmal cough, post-tussive emesis, decreased nasal congestion, absence of fever, cyanosis, and acute life-threatening events such as apnea or seizures. Notably, 16% of pertussis cases were found to have co-infections with other respiratory pathogens.^9^

### Blood workups

#### Complete blood count

Leukocytosis and lymphocytosis have a strong correlation with the prognosis of pertussis, especially in infants. These conditions may be absent in the catarrhal phase but tend to be high during the paroxysmal stage. This is attributed to the action of pertussis toxin that triggers leukocytosis. In severe cases, hyperleukocytosis may exceed 100 × 10^3^/μL and is associated with fatal complications in infants.^10^

#### Culture of nasopharyngeal secretions

The traditional gold standard for diagnosing pertussis is the culture of nasopharyngeal secretions. However, obtaining results can take up to a week. While culture is 100% specific in identifying *Bordetella pertussis*, its sensitivity is low and decreases even further after the first two weeks of coughing.^11^

#### Polymerase chain reaction of nasopharyngeal secretion

The PCR demonstrates a high sensitivity for detecting pertussis, particularly within three weeks following the onset of cough. Due to the abundance of IS481 insertion sequences (over 240 copies) in the genome of *Bordetella pertussis*, PCR kits mainly target this sequence for a higher sensitivity. However, since IS481 may also be present in other *Bordetella* species, additional targets, such as the single-copy pertussis toxin promotor target (ptxP), can be included to specifically identify *Bordetella pertussis*. Although this test is both specific and timely, it is not suitable for screening asymptomatic individuals or their contacts due to the risk of transient colonization or contamination, which may lead to false positive results. Therefore, meticulous care must be taken during sample collection and laboratory processing to prevent contamination.^3,12,13^

Recent commercially available PCR panels are able to detect multiple respiratory pathogens from a single specimen, including *Bordetella pertussis*, thereby leading to higher detection of co-infections.^14,15^ Variations of PCR techniques, such as nested PCR and multiplex PCR, are often used. Nested PCR consists of two amplification steps: first, an outer primer amplifies a larger gene segment, increasing sensitivity. Then, an inner primer amplifies a smaller region within the previous product, enhancing specificity. In contrast, multiplex PCR simultaneously amplifies multiple genes in a single reaction.^16^ In our study, the PCR specimens were analyzed using one of the two commercially available multiplex nested PCR panels (GenMark ePlex and BioFire FilmArray Torch).

#### Serological detection of antibodies

This method can detect pertussis up to 12 weeks following the onset of cough. However, its application is limited due to heterogeneity between laboratories and low specificity, which can arise from the effect of previous vaccinations and interaction with other *Bordetella* species.^3,11,13^

### Radiologic findings

Chest radiographs typically appear normal in uncomplicated cases. A study conducted by Bellamy et al. estimated that 26% of children hospitalized with whooping cough had abnormal chest radiographs. These abnormalities were more common in patients older than one year. Pulmonary consolidation was the most common abnormality (21%), with peribronchial consolidation being the most common prevalent type (72%). Lymphadenopathy was observed in 9% of patients, while lobar collapse was observed in 4%. Follow-up radiographs did not reveal any significant residual abnormalities.^17^

### Management

#### Hospital admission

Patients exhibiting complications such as respiratory distress, apnea, cyanosis, seizures, and poor oral intake require hospitalization.

Additionally, due to the increased risk of morbidity and mortality in infants under four months of age, it is recommended that these patients be admitted to a hospital equipped with a PICU.^18^ Droplet precautions are advised for five days after initiating antibiotic therapy or for three weeks after the onset of coughing fits if antibiotics have not been administered.^19^

#### Supportive care

The administration of intravenous fluids or feeding via a nasogastric tube is essential for ensuring adequate hydration and maintaining normal nutritional status. Since coughing fits can be exhaustive to patients, it is crucial to avoid exposure to cough triggers such as cold temperature, excessive suctioning, or exercising as much as possible. Research indicates that antitussive medications, steroids, antihistamines, and bronchodilators do not provide significant relief for pertussis cough.^20^

#### Antibiotic therapy

In cases where pertussis is suspected, antimicrobial therapy should be started before obtaining laboratory confirmation. Administering antibiotics during the early phases of pertussis may reduce the duration of symptoms. This antimicrobial therapy eradicates nasopharyngeal bacteria, thereby decreasing the risk of disease transmission. Antibiotics are recommended for infants and children exhibiting symptoms that began within the past three weeks, as well as for infants under one year of age who have tested positive via PCR or culture within six weeks of the onset of cough.^21^

Macrolides are the first-line therapy, with azithromycin administered daily for five days being the preferred agent for infants under one month of age. Other macrolides, such as clarithromycin, given twice daily for seven days, and erythromycin, administered four times daily for 14 days, can be used for children older than one month. Short-term treatments with azithromycin or clarithromycin have demonstrated comparable efficacy to long-term erythromycin lasting 10–14 days, while also resulting in fewer side effects. However, despite the advantages of macrolide therapy, its use during the first month of life has been associated with an increased risk of developing infantile hypertrophic pyloric stenosis.^21,22^

Trimethoprim-sulfamethoxazole serves as an alternative agent when allergy or bacterial resistance prevents macrolide administration.^21^

### Prevention

#### Routine vaccination for pertussis

The DTaP vaccine (containing diphtheria, tetanus, and acellular pertussis) decreases the risk of severe pertussis in infancy. The World Health Organization recommends administering three primary doses of this vaccine, with the first dose commencing as early as six weeks of age, followed by two doses given at intervals of over four to eight weeks. A booster dose is recommended during the second year of the child's life, with additional boosters potentially required later based on local epidemiology.^23^

#### Maternal vaccination

Administering the Tdap vaccine during pregnancy is considered safe and provides protection to infants during their first months of life through the passive transfer of maternal antibodies. This recommendation was first established in the USA in 2011, and it is now endorsed by over 40 countries. The recommended timing for maternal Tdap vaccination is between 27 and 36 weeks of gestation. To maximize the maternal antibody response and passive antibody transfer to the newborn, it is recommended to administer the vaccine as early as possible in the gestation period of 27–36 weeks. However, the Tdap vaccine can be safely given at any stage of pregnancy when necessary for managing wounds, responding to pertussis outbreaks, or addressing other extenuating circumstances.^24^ A systematic review conducted by Kandeil et al. showed that the effectiveness of maternal Tdap vaccination in preventing pertussis in infants below three months of age ranged between 69% and 93%. Furthermore, it was found to prevent pertussis-associated hospitalizations by 58.3–94% for infants up to six months of age.^8^

The Ministry of Public Health in Qatar advocates for Tdap vaccination among pregnant women. However, the exact rate of maternal vaccination coverage is not clearly established, hindered by hesitancy among pregnant women and a lack of public awareness about the risks of pertussis in infants.

## Conclusion

Infants, particularly those under three months of age, are more susceptible to pertussis infection and face an elevated risk of severe complications. To mitigate this risk, administering vaccines to pregnant women in the third trimester has proven to be an effective strategy. This approach fosters maternal immunity, thereby offering passive protection to newborns and young infants.

Although vaccination during pregnancy is a crucial preventive measure, further research is needed to assess the factors influencing vaccine uptake among pregnant women. Understanding the attitudes and concerns of both healthcare providers and expectant mothers is essential for optimizing vaccination rates by addressing potential barriers and promoting the benefits of maternal vaccination.

### List of abbreviations

[Table tbl2]

### Ethical approval and consent for publication

The study was reviewed and approved by the Institutional Review Board of Hamad Medical Corporation (Protocol ID: MRC-04-24-546).

### Competing interests

The authors declare that they have no competing interests.

## Figures and Tables

**Table 1.** Summary of the key characteristics of the patients included in the case series.

	First presentation age	Sex	Baby DTaP vaccination status	Maternal Tdap pregnancy vaccination status	Number of encounters before diagnosis	Admission days in the pediatric ward	Admission days in the PICU	Associated viral infections

Case 1	1 month and 20 days	Female	Not vaccinated	Not given	2	1	0	Mycoplasma pneumonia, respiratory syncytial virus

Case 2	2 months and 13 days	Male	5 days before the first presentation	Not given	2	3	0	Human rhino, enterovirus

Case 3	1 month and 9 days	Female	Not vaccinated	Not given	2	2	8	Human rhino, enterovirus

Case 4	1 month and 27 days	Male	Not vaccinated	Not given	3	5	0	No


**Table tbl2:** 

COVID-19	Coronavirus Disease 2019

DTaP/Hib/IPV/HBV	Diphtheria, Tetanus, acellular Pertussis/Hemophilus influenzae type b/Inactivated Poliovirus/Hepatitis B Vaccine

IS481	Insertion Sequence 481

PCR	Polymerase Chain Reaction

PICU	Pediatric Intensive Care Unit

PtxP	Pertussis toxin promotor target

Tdap	Tetanus, Diphtheria, acellular Pertussis Vaccine

WBC	White Blood Cell

